# IL-1 in osteoarthritis: time for a critical review of the literature

**DOI:** 10.12688/f1000research.18831.1

**Published:** 2019-06-21

**Authors:** Tonia L. Vincent

**Affiliations:** 1 Centre for OA Pathogenesis Versus Arthritis, Kennedy Institute of Rheumatology, University of Oxford, Oxford, OX3 7FY, UK

**Keywords:** Interleukin-1, catabolin, osteoarthritis, cartilage

## Abstract

The concept of interleukin-1 (IL-1) as a target in osteoarthritis (OA) has been an attractive one for many years. It is a highly potent inducer of cartilage degradation, causing the induction of mRNA and controlling the bioavailability of disease-relevant proteases such as ADAMTS5 and MMP13. It drives synovitis and can induce other disease-relevant genes such as nerve growth factor, a key pain sensitiser in OA. However, the quality of evidence for its involvement in disease is modest. Descriptive studies have demonstrated expression of IL-1α and β in OA cartilage and elevated levels in the synovial fluid of some patients. Agnostic transcriptomic and genomic analyses do not identify IL-1 as a key pathway. *In vivo* models show a conflicting role for this molecule; early studies using therapeutic approaches in large animal models show a benefit, but most murine studies fail to demonstrate protection where the ligands (IL-1α/β), the cytokine activator (IL-1–converting enzyme), or the receptor (IL-1R) have been knocked out. Recently, a number of large double-blind randomised controlled clinical studies targeting IL-1 have failed. Enthusiasm for IL-1 as a target in OA is rapidly dwindling.

## Introduction

Evidence to support a molecular role in disease is usually amassed from a combination of biological plausibility, demonstration of disease-relevant activity *in vitro*, descriptive studies in disease tissue samples and genetic analyses. The quality of evidence is improved when it is possible to perform functional validation studies in relevant animal models in which the molecule is knocked down (or out) or to use drugs that target the molecule in question. Ultimately, definitive clinical studies will reveal the success of one’s endeavours but with the caveat that you have selected your patient group appropriately and have sensitive and relevant outcome measures. In practice, it is hard to build a robust case to justify the large investment required to support a clinical trial and this is further complicated by publication bias and uncritical analysis. In this commentary, I review the evidence for interleukin-1 (IL-1), arguably the best-investigated cytokine in osteoarthritis (OA) pathogenesis, and take a critical look at the quality of evidence that supports its role in disease. This is not intended to be an exhaustive review of all the literature on IL-1 in OA. Rather, I have selected key articles that add substantially to our knowledge irrespective of whether they report ‘positive’ or ‘negative’ outcomes.

## Interleukin-1, the history

The term ‘IL-1’ was first ascribed to a potent cytokine activity that was generated by activated macrophages, and shown to activate T cells ^1– 3^. This molecule was initially termed ‘lymphocyte-activating factor’ but was reclassified to IL-1 in 1979 ^4^. Over the following 15 years, a number of other cell activities were ascribed to IL-1. These included ‘mononuclear cell factor’ ^5^ and ‘endogenous (or leukocytic) pyrogen’, the latter being able to induce fever by direct stimulation of the hypothalamus ^6, 7^. Immune cells were not the only focus of this early work. Connective tissue cells were also shown to make and respond to an IL-1–like activity ^8, 9^, the most striking of which was termed ‘catabolin’ ^10^ ( Table 1).

**Table 1.** Cellular activities eventually attributed to IL-1.

Interleukin-1 (IL-1) synonym	Role
Leukocyte-activating factor	T-cell activation
Mononuclear cell factor	IL-2 induction
Catabolin	Cartilage degradation Synovitis
Endogenous pyrogen/ Leukocyte pyrogen	Fever

## Biological plausibility

Catabolin was initially described in co-cultures of synovium with articular cartilage ^11^ and subsequently purified and characterised from non-adherent porcine leukocytes (principally lymphocytes) ^10, 12– 14^. It caused profound loss of proteoglycan from cartilage *in vitro* and *in vivo* which was deemed to be due to catabolic activity, as well as suppression of new proteoglycan synthesis ^15, 16^. The catabolic activity that was demonstrated in chondrocytes was indirect as dead cartilage was non-responsive to catabolin, and it was postulated that catabolin induced the enzymes responsible for aggrecan degradation. For a time, there was a consensus that this IL-1–like activity was not IL-1 as the isoelectric point (pI) of catabolin was acidic (4.8–5.0) compared with IL-1, which had a pI of between 6.5 and 7. Subsequent purifications by several groups in mouse, human and pig revealed two distinct molecular species, which have similar biological activities ^17^. When these molecules were eventually cloned, they turned out to have only modest amino acid sequence homology (20%) and distinct pIs. They were termed IL-1α and IL-1β, reflecting the acidic and basic proteins respectively ^17, 18^. Both ligands bound to the same two-chain receptor (IL-1R) with high affinity (k _d_ = 10 ^−10^) ^19, 20^. Another molecule with some shared homology with IL-1α and IL-1β was discovered. This turned out to be the IL-1R antagonist (IL-1Ra), a natural inhibitor of IL-1 signalling ^21– 23^.

IL-1α and IL-1β are made as pro-proteins which lack signal peptides and are retained in the cytoplasm ^24^. Unlike pro–IL-1α, pro–IL-1β lacks biological activity and must be processed by caspase 1, also known as IL-1–converting enzyme (ICE) ^25, 26^. Processing by caspase 1 is linked to secretion of IL-1β and this therefore is a requirement for its biological effects. IL-18 is processed in a similar fashion. These effects are dependent upon activation of the ‘inflammasome’ complex and indicate that IL-1 biological activity requires a two-hit process: induction of the mRNA followed by processing of the pro-molecule prior to secretion from the cell ^27^.

## *In vitro* disease-relevant activity

IL-1 was hugely influential in the discovery of the proteases responsible for cartilage degradation in OA. IL-1 was able to induce a number of known matrix metalloproteinases (MMPs) *in vitro* and suppress proteoglycan synthesis ^9, 16, 28– 30^ but it was not until the large-scale purification of medium from IL-1–stimulated cartilage that the first aggrecanase (a disintegrin with thrombospondin motif 4, or ADAMTS4) was identified ^31^. By homology searching, this led to the identification of ADAMTS5 ^18^. The role of ADAMTS5, but not ADAMTS4, in OA pathogenesis was subsequently shown by Glasson *et al*. in mice (2005) ^32, 33^. There are conflicting views on whether ADAMTS5 and ADAMTS4 are both pathogenic mediators in human OA ^21, 23^. Interestingly, in most species, ADAMTS5 is constitutively expressed and is not much regulated by IL-1 at the mRNA level ^34^, even though its activity is strongly IL-1–regulated ^21^. Regulation of activity is thought to be controlled by the re-uptake of ADAMTS5 at the cell surface by the scavenger receptor LRP1 ^35, 36^. It is worth noting that IL-1 is used as an exemplar in these studies and is ideally suited because of its potency and understood mechanisms of action. Other cytokines, including tumour necrosis factor (TNF) ^37^, retinoic acid ^34, 38^ and oncostatin M ^39^, also are strong inducers of cartilage catabolism *in vitro*.

## Interleukin-1 regulation in human osteoarthritis tissues

A standard initial approach to validation of candidate molecules in disease almost always involves the demonstration that the molecule is upregulated in diseased tissues. This is challenging in OA in particular because of difficulties in obtaining normal tissue as a comparator. IL-1 is highly potent and usually present at very low concentrations. It is not easy to detect by conventional enzyme-linked immunosorbent assay or even by higher-sensitivity assays such as the MesoScale Discovery platform ^40^. However, using high-sensitivity assays, some groups have detected low levels (<1 pM) of IL-1 in the synovial fluid of some patients with OA and rarely in normal joints ^41^. There are studies reporting positive immunohistochemistry for IL-1α and IL-1β and ICE as well as *in situ* hybridisation data for ICE in established human OA cartilage ^42, 43^. However, these studies compare expression levels within OA tissue and do not compare with normal tissue. These controls are especially important when we consider that IL-1 is made as a pro-enzyme and that intracellular staining does not correlate with secretion and activity. Moreover, work from our group previously showed that simple mechanical injury (that occurs at the time of tissue dissection) is a strong inducer of pro–IL-1 in normal healthy tissue and could easily confound the analysis ^44^ ( Table 2).

**Table 2.** Evidence for a role of IL-1 in osteoarthritis.

Type of evidence	Quality of evidence (likely role in disease)
Biological plausibility	High
*In vitro* disease-relevant activity	High
Human osteoarthritis tissue studies	Low (not identified by agnostic ‘-omic’ analyses)
Pre-clinical knockout studies	Moderate (conflicting role in disease)
Human clinical studies	High (little role in disease)

Counting the IL-1–positive cells within the synovium of patients with OA and rheumatoid arthritis (RA) demonstrates that OA synovial cells are much less likely to be positive (20%) than RA cells (60%) ^6, 7, 45^. In several similar studies, OA tissue is generally regarded as the negative control, so again normal tissue is not included.

## Molecular studies

Microarray studies potentially allow one to examine regulated genes in disease in an unbiased fashion. Early array studies in OA cartilage did not demonstrate elevation of *IL-1* mRNA in OA compared with normal cartilage ^46^ or in lesional compared with non-lesional OA tissue ^47^. In a larger study by Aigner *et al*., who studied 4000 genes in 78 patient and control samples, *IL-1* was downregulated in disease by around 50% ^48^. Two recent RNA sequencing (RNA-Seq) analyses deserve special mention. One study, by Soul *et al*., performed RNA-Seq on the articular cartilage of patients undergoing knee replacement surgery and compared gene expression with non-disease cartilage in the same joint ^49^. An unbiased analysis identified two molecularly distinct groups within the affected OA samples. Pathway analysis revealed over-representation of complement activation pathways, innate immune responses, Wnt and transforming growth factor beta (TGFβ) signalling. There was a notable absence of an inflammatory cytokine signature ^49^. The first single-cell RNA-Seq study in OA articular cartilage was recently published ^50^. Although this study did not have normal cartilage as a comparator, *IL-1* did not feature as a marker for one of the seven phenotypically distinct groups of OA chondrocytes. Nor was it associated with a molecular signature that predicted disease progression ^50^.

## Genetics

There are replicated candidate studies in which polymorphic variants of *IL-1* have been shown to be increased in OA compared with a non-OA population (reviewed in 51), but *IL-1* has not come out of any of the genome-wide association studies which have looked agnostically across the genome either by mapping polymorphic variants or by whole genome sequencing. The largest of these studies, recently published by the Zeggini group, identified 64 disease loci (52 of them novel) from over 77,000 large-joint OA cases ^52^. Of these, four strong groups emerge: (1) *TGFβ* family members, including candidate genes *GDF5, TGFβ1, LTBP1, LTBP*3 and *SMAD3*; (2) *TGFα*, which has strong independent pre-clinical data to support it as a target ^53^; (3) fibroblast growth factors (FGFs), in particular *FGF18* and its receptor *FGFR3*; FGF18, is showing significant promise following intra-articular injection in clinical trial ^54, 55^ and (4) *ALDH1A2*, encoding the enzyme that synthesises retinoic acid, a strong genome-wide association study hit in hand OA ^56^ and now in knee. Again, the absence of an inflammatory cytokine signature is noteworthy.

## Pre-clinical studies

Prior to the genetic modification era, pre-clinical OA was largely restricted to large animals (for example, dog and rabbit). A few studies looked at therapeutic targeting of IL-1 using either recombinant IL-1Ra (anakinra) or gene transfer of IL-1Ra. All of these early studies showed striking protection in rapidly progressive surgical models of OA when treatment was initiated early after surgery ^57– 59^. Similar protection was seen in rats after anterior cruciate ligament transection when treated early with recombinant intra-articular IL-1Ra ^60^. D’Lima *et al*. showed that caspase 1 inhibition suppressed disease in rabbits after cruciate ligament transection when delivered three times per week for 9 weeks ^61^. The latter study could be affecting molecules other than IL-1, such as IL-18.

The first surgical models of OA in genetically modified mice were performed in 2003 by Clements *et al*. ^62^. In that study, partial meniscectomy was performed in four genetically modified strains: *Il1b* knockout, *Ice* knockout, *Mmp3* knockout and nitric oxide synthase ( *Nos*) knockout mice. None of these strains demonstrated reduced disease; if anything, a modest increase in disease was observed ^62^. Kawaguchi’s group also reported (although data were not included in the article) a lack of protection in *Il1a/Il1b* double-knockout mice ^63^. Our own group has failed to see protection in *Il1r1* knockout mice (unpublished data). Only one report of protection in the *Il1b* knockout mouse has been published and this was in a review article ^64^, in which a 40% reduction in disease at one time point (8 weeks) was observed. The number of animals used in this experiment was not specified.

## Human clinical studies

One assumes that positive results in small open-label clinical studies ^65^ are partly responsible for driving the decision to proceed to randomised controlled trials (RCTs) in OA using IL-1 targeting therapies. These have included, most recently, two large studies by Abbvie using a dual neutralising antibody against IL-1α and IL-1β in hand OA ^66^ and knee OA ^67^. Both studies failed to reach their primary outcome target and concluded lack of efficacy. Similarly, a single intra-articular injection of anakinra failed to show clinical efficacy at 3 months, the primary endpoint, in an RCT of 160 individuals with knee OA ^68^. A randomised double-blind controlled study of an IL-1R neutralising antibody also failed to meet the primary endpoint ^69^. Two small studies, one open-label and one placebo-controlled, demonstrated reduction in pain in individuals after knee trauma with IL-1Ra (anakinra). It is important to stress that whilst knee trauma may lead to OA over the course of 5 to 10 years in 50% of cases ^70^, there is no evidence that early pain after injury is indicative of OA or that inhibiting IL-1 early after injury can prevent OA developing ^71, 72^.

### Co-existing crystal arthropathy

Could IL-1 still have a role in a subset of patients with OA? One reasonable hypothesis is that some patients’ disease may be complicated by crystal arthritis. Calcium pyrophosphate (CPP) and basic calcium phosphate (BCP) crystals are present in the synovial fluid of around 20 to 25% of patients with knee OA ^73^ and CPP in 13% of patients with small-joint (hand and wrist) OA ^74^. Although the presence of crystals in the joint does not necessarily lead to a clinical crystal arthritis, all crystals (including urate, pyrophosphate and cholesterol) are potential activators of the inflammasome pathway, resulting in caspase 1–dependent processing of intracellular pro–IL-1 ^75– 77^. In one study, urate levels within the synovial fluid correlated strongly with IL-1β (in those samples in which it was measurable) and IL-18 ^41^. Radiographic disease severity correlated with levels of IL-1 ^41^. These results identify synovial fluid urate or IL-1/IL-18 levels (or both) as potential biomarkers of disease severity. The jury is still out on whether IL-1 may be driving pathogenesis in a small subgroup of OA individuals who have an active crystal arthritis contributing to their structural and symptomatic disease ^78^. In view of the complete lack of signal from the anti–IL-1 clinical trials, one has to assume that the proportion of such individuals within the larger OA population is small.

## Conclusions

IL-1 remains the most potent inducer of cartilage degradation we know of and from the early days of ‘catabolin’ has been a top molecular candidate in OA. In recent years, IL-1 has proven to be a good target in diseases due to genetic defects in the inflammasome pathway, including some of the rare periodic fever syndromes, and this has been a good opportunity to validate the available therapies ^79^. IL-1 targeting is also efficacious and licenced for use in crystal arthritis ^80^, although it is usually reserved for those with severe disease unresponsive to first-line treatments. Randomised clinical trials in OA are conclusively telling us that this is not a target in OA despite all our hopes. Could this have been anticipated earlier? In retrospect, the evidence for IL-1 from the clinical data, especially those acquired from agnostic whole genome and whole transcriptome analyses, was weak. Results from pre-clinical studies were polarised in their conclusions and this calls into question the robustness of these data. Failure to reduce sources of bias e.g. through randomisation of animals, blinding experimentor to treatment group, or double blind scoring, are rarely described and probably not often performed. Studies are often under-powered and may examine outcomes at only one time point. Animal studies in particular are further confounded by a publication bias towards positive results ^81^. RCTs are an expensive way to disprove a major role for a molecule in disease, but the journey nonetheless brings us closer to understanding OA pathogenesis.
